# Prostate cancer patients’ self-reported participation in research: an examination of racial/ethnic disparities

**DOI:** 10.1007/s10552-021-01463-9

**Published:** 2021-06-29

**Authors:** Nynikka R. Palmer, Hala T. Borno, Steven E. Gregorich, Jennifer Livaudais-Toman, Celia P. Kaplan

**Affiliations:** 1 Department of Medicine, Division of General Internal Medicine at Zuckerberg, San Francisco General Hospital, University of California San Francisco, UCSF, 1001 Potrero Avenue, Box 1364, San Francisco, CA 94143 USA; 2 Helen Diller Family Comprehensive Cancer Center, University of California San Francisco, San Francisco, USA; 3 Department of Medicine, Division of Hematology/Oncology, University of California San Francisco, San Francisco, USA; 4 Department of Medicine, Division of General Internal Medicine, University of California San Francisco, San Francisco, USA; 5 Multiethnic Research Center, University of California San Francisco, San Francisco, USA

**Keywords:** Prostatic neoplasm, Healthcare disparities, Minority groups, Men, Research subjects

## Abstract

**Purpose:**

We examined prostate cancer patients’ participation in research and associated factors by race/ethnicity in a multiethnic sample.

**Methods:**

Men with a new diagnosis of prostate cancer were identified through the California Cancer Registry. Patients completed a cross-sectional telephone interview in English, Spanish, Cantonese or Mandarin. Multivariable logistic regression models, stratified by race/ethnicity, estimated the associations of patient demographic and health characteristics with participation in (1) any research, (2) behavioral research, and (3) biological/clinical research.

**Results:**

We included 855 prostate cancer patients: African American (19%), Asian American (15%), Latino (24%), and White (42%). In the overall model of participation in any research, African American men (Odds Ratio (OR) = 2.54, 95% CI 1.63–3.94), and those with two or more comorbidities (OR = 2.20, 95% CI 1.27–3.80) were more likely to report participation. Men 65 years old and older (OR = 0.65, 95% CI 0.47–0.91), those who were married or living with a partner (OR = 0.67, 95% CI 0.45–0.98), and those who completed the interview in Spanish (OR = 0.36, 95% CI 0.15–0.85) were less likely to report participating in any research. Stratified analyses identified racial/ethnic-specific sociodemographic characteristics associated with lower research participation, including Spanish or Chinese language, older age, and lower education.

**Conclusion:**

African American prostate cancer patients reported higher research participation than all other groups. However, recruitment efforts are still needed to overcome barriers to participation for Spanish and Chinese speakers, and barriers among older adults and those with lower education levels.

## Introduction

In the United States, prostate cancer (PCa) remains the second leading cause of cancer-related deaths among men [1, 2]. Racial/ethnic disparities in health outcomes, quality of life, and access to treatment among men with PCa are observed on the population level [3]. Emerging literature describes the complex and multifaceted drivers of these observed disparities and the interaction between access to care, quality of care, and genetic and environmental factors [4]. Additionally, health services research has delineated differential access to care that may add to the disparities in outcomes [5]. Differences in culture, attitudes, behaviors, and preferences have also been observed to contribute to variation in PCa outcomes [6]. Lastly, it is well established that the underrepresentation of racial/ethnic minorities in clinical research, compared to White participants, may exacerbate these disparities [7, 8]. To understand racial/ethnic disparities among men with PCa, it is essential to examine all aspects of the cancer care continuum from prevention, screening/early detection, diagnosis, treatment, to survivorship. Participation in all forms of research (e.g., behavioral, biological, clinical trials) is vital to mitigate disparities across this care continuum. Accruing underrepresented racial/ethnic minorities in all aspects of cancer research better ensures that novel therapies and interventions are effective, treatment options are communicated in a culturally and linguistically appropriate manner, and behavioral interventions are acceptable and relevant for all men affected by the disease [9].

A vast literature exists characterizing the problem of racial/ethnic disparities in therapeutic clinical trials research as well as the attitudes and beliefs associated with participation in investigational drug or novel treatment regimens [10, 11]. However, there is limited research on participation rates of racial/ethnic minorities compared to White participants in other types of research such as behavioral/prevention studies. By extension, to date, the factors associated with racial/ethnic minority participation in different types of clinical and behavioral research are not well understood.

This study adds to an important area of PCa disparities literature by investigating participation in different types of research among a multiethnic sample. Specifically, in this study, we examined (1) racial/ethnic differences in reported participation in behavioral and biological research and (2) characterized the types of research previously participated in. Given the need for representative study populations in all aspects of research in order to achieve equity in health outcomes [12], this study aims to understand if disparities in minority clinical research participation persist when examined by study type.

## Methods

Men diagnosed with PCa in 2008 were recruited from the Greater San Francisco Bay Area and Los Angeles County to participate in a cross-sectional telephone interview between November 2011 and November 2012. All participants were identified from the California Cancer Registry case-listings, a statewide population-based surveillance system.

### Eligibility criteria

Participants’ eligibility criteria included (a) self-identification as Black/African American, Latino, Asian American or non-Latino White, (b) diagnosed with stage I and II, localized PCa in 2008, (c) between 18 and 75 years old, (d) speaks Cantonese or Mandarin, Spanish, or English, (e) patients’ physician did not object to their participation, and (f) no physical, cognitive or mental disability.

All minority men (African American, Latino, and Asian American) and a random sample of the White men with PCa who met the study criteria were recruited to participate in this study. Letters were mailed to the physicians listed in the California Cancer Registry to identify potential participants who should not be contacted. One month after the physician letters were mailed, eligible participants (those whose physician did not object to their participation) received an introductory recruitment letter in English, Spanish, or Chinese with opt-out options. Our team followed a strict protocol of up to six telephone calls to reach eligible participants. Study participants provided verbal consent by telephone prior to participation. Bilingual interviewers conducted 30-min telephone interviews in the participant’s preferred language. Further details outlining study methods have been described previously [13, 14]. All study activities were approved by the University of California, San Francisco Institutional Review Board (#10-00858) and the California State Committee for the Protection of Human Subjects (#2018-164).

### Data measures

#### Descriptive variables

The study collected the following demographic information: age (categorized as 40–54 years; 55–64 years, ≥ 65 years); self-reported race/ethnicity (Black/African American, Asian American, Latino or non-Latino White); marital status (married or living with a partner vs. other); educational level (high school or less, some college, college degree or beyond); California region (Northern California vs. Southern California); language of interview (English, Spanish, Cantonese or Mandarin); and health insurance coverage (any private vs. public, government or no insurance). If participants reported more than one racial/ethnic group, they were asked to identify which race/ethnicity they most identified with, and we used that response for their self-reported race/ethnicity. For anyone who did not answer the race/ethnicity question, we used the race/ethnicity recorded in the cancer registry. Health literacy was assessed using a validated scale including three questions assessing (a) "How often do you have someone like a family member, friend, hospital worker or caregiver, help you read hospital material;” (b) “How often are you uncomfortable filling out medical forms by yourself?;” and (c) “How often do you have problems learning about your medical condition because of difficulty understanding the written information?” Response categories ranged from 1 = always to 5 = never [15]. A composite health literacy score was created averaging the responses of the three questions. Based on this score, a dichotomous variable for health literacy was created (low health literacy = score < 3.5; medium to high health literacy = score ≥ 3.5).

#### Health-related indicators

Health status was assessed using one question, *how would you rate your health*, which was dichotomized into excellent/very good versus good/fair/poor [16]. Participants were asked if they were ever told by a doctor that they had heart disease, high blood pressure, lung disease, diabetes, ulcer or stomach disease, kidney disease, liver disease, other cancer, depression, arthritis, and other health conditions [17]. Responses were categorized into 0, 1, and 2 or more reported comorbidities. The Gleason score (a grading system for PCa tumors) derived from the California Cancer Registry, was dichotomized as 1–6 versus 7–10. PCa treatment modality was self-reported and included active surveillance/watchful waiting, surgery, brachytherapy, external radiation, hormone therapy, chemotherapy, and any other treatment. Treatment was further dichotomized as having received treatment with or without chemotherapy or hormone therapy, as first line of treatment which would indicate greater disease severity.

#### Outcomes

The survey assessed whether participants reported experience in a health research study, before or after the PCa diagnosis. Four questions specifically inquired about prior research participation in (a) surveys, interviews, or focus groups; (b) collection of blood or tissue samples; (c) clinical trials (e.g., new medicine, medical treatment or procedures; and (d) behavioral change interventions (e.g., diet or exercise). We created four categories of research participation: any, behavioral, biological/clinical, and none. Any research participation was created by a global indicator of participation in any of the research reported above. We combined surveys, interviews, focus groups, and behavioral interventions into behavioral research. And biological/clinical research included the collection of blood or tissue samples and clinical trials.

### Statistical analysis

Descriptive statistics were used to summarize study participants’ demographic and health-related characteristics, and to examine differences in demographic characteristics by the three outcomes: participation in any research, behavioral research, or biological/clinical research. Multivariable logistic regression models were fit, reporting odds ratios and 95% confidence intervals (95% CI), in which each model adjusted for age, marital status, education, California region, language of interview, insurance, health literacy, health status, comorbidities, Gleason score, and treatment with or without chemotherapy. Overall adjusted models pooled data across all four racial/ethnic groups and contrasted each minority group with White participants. We also stratified models by race/ethnicity to identify specific factors associated with outcomes within each racial/ethnic group. All statistical analysis were conducted using STATA/SE Version 14.0. The p-value for statistical significance was set to < 0.05.

## Results

Study participant characteristics, stratified by participation in research, are listed in Table 1. The analytic sample (*n* = 855) consisted of 19.2% African American (*n* = 164), 14.7% Asian American (*n* = 126), 24.1% Latino (*n* = 206), and 42% non-Latino White (White; *n* = 359) men with a history of PCa who completed the survey (45% response rate, 855/1,890). As reported in prior research [13, 14], racial/ethnic differences were noted across all demographic and health-related characteristics. We found statistically significant differences within participation in any, behavioral, and biological/clinical research by various sociodemographic and clinical characteristics. For any research, participants who were African American, less than 64 years old, single/divorced/widowed, had some college education, completed the survey in English, had higher health literacy, or two or more comorbidities reported higher participation. For behavioral research, we found similar significant differences, with the addition of participants who had excellent/very good health status reporting higher participation. Similar significant differences were also noted for biological/clinical research participation, with a slight difference in college graduate or beyond (versus some college education) reported the highest level of participation. We found no significant differences across region, health insurance, Gleason score, or treatment received.

**Table 1:** Study participant characteristics by participation in research (*n* = 855)

	Total	Any research	*p*-value	Behavioral	*p*-value	Biological/clinical	*p*-value
	*N* (%)	*N* (%) Yes		*N* (%) Yes		*N* (%) Yes	
Race			< 0.001		< 0.001		< 0.001
African American	164 (19.2)	78 (47.6)		60 (36.6)		57 (34.8)	
Asian American	126 (14.7)	21 (16.7)		14 (11.1)		15 (11.9)	
Latino	206 (24.1)	35 (17.0)		27 (13.1)		16 (7.7)	
White	359 (42.0)	94 (26.2)		77 (21.5)		58 (16.1)	
Age			< 0.001		0.025		0.008
40–64 years	403 (47.6)	131 (32.5)		98 (24.3)		84 (20.8)	
≥ 65 years	444 (52.4)	97 (21.9)		80 (18.0)		62 (14.0)	
Marital status			0.001		< 0.001		0.002
Single/divorced/widowed	204 (24.2)	73 (35.8)		62 (30.4)		50 (24.5)	
Married/living with partner	638 (75.8)	153 (24.0)		114 (17.9)		95 (14.9)	
Education			0.006		0.006		0.018
High school or less	214 (25.7)	40 (18.7)		29 (13.6)		24 (11.2)	
Some college	193 (23.1)	59 (30.6)		49 (25.4)		35 (18.1)	
College graduate or beyond	427 (51.2)	126 (29.5)		97 (22.7)		86 (20.1)	
Region			.769		.799		.383
Northern California	502 (58.7)	132 (26.3)		106 (21.1)		81 (16.1)	
Southern California	353 (41.3)	96 (27.2)		72 (20.4)		65 (18.4)	
Language of interview			< 0.001		< 0.001		< 0.001
English	710 (83.0)	216 (30.4)		171 (24.1)		142 (20.0)	
Spanish	109 (12.8)	10 (9.2)		7 (6.4)		2 (1.8)	
Cantonese/Mandarin	36 (4.2)	2 (5.6)		0		2 (5.6)	
Health Insurance			.252		.201		.554
Private	663 (79.9)	184 (27.8)		145 (21.9)		116 (17.5)	
Public/government/none	167 (20.1)	39 (23.4)		29 (17.4)		26 (15.6)	
Health literacy			< 0.001		0.001		0.001
Low (< 3.5)	161 (19.2)	25 (15.5)		19 (11.8)		13 (8.1)	
Medium to high (≥ 3.5)	676 (80.8)	202 (29.9)		158 (23.4)		132 (19.5)	
Health status			0.091		0.027		0.433
Good/fair/poor	423 (49.5)	102 (24.1)		75 (17.7)		68 (16.1)	
Excellent/very good	431 (50.5)	126 (29.2)		103 (23.9)		78 (18.1)	
Number of comorbidities			0.021		0.014		0.016
0	119 (14.0)	20 (16.8)		13 (10.9)		13 (10.9)	
1	196 (23.0)	51 (26.0)		42 (21.4)		26 (13.3)	
2 or more	537 (63.0)	157 (29.2)		123 (22.9)		107 (19.9)	
Gleason score			0.677		0.680		0.922
1–6	369 (43.5)	101 (27.4)		79 (21.4)		63 (17.1)	
7–10	479 (56.5)	125 (26.1)		97 (20.3)		83 (17.3)	
Prostate cancer treatment with hormone therapy or chemotherapy			0.391		0.591		0.927
Yes	130 (15.4)	39 (30.0)		25 (19.2)		22 (16.9)	
No	713 (84.6)	188 (26.4)		152 (21.3)		123 (17.3)	

Number of missing responses: *n* = 8 (age); *n* = 13 (marital status); *n* = 21 (education); *n* = 0 (region, language of interview); *n* = 25 (insurance); *n* = 18 (health literacy); *n* = 1 (health status); *n* = 3 (comorbidities); *n* = 7 (Gleason score); *n* = 12 (hormone therapy)

### Multivariable analysis

In the overall model (Table 2) for participation in any research, African American men (OR = 2.54, 95% CI 1.63–3.94) and those with two or more comorbidities (OR = 2.20, 95% CI 1.27–3.80) were more likely to report participation. Men 65 years old and older (OR = 0.65, 95% CI 0.47–0.91), those who were married or living with a partner (OR = 0.67, 95% CI 0.45–0.98), and those who completed the interview in Spanish (OR = 0.36, 95% CI 0.15–0.85) were less likely to report participating in any research.

**Table 2:** Adjusted ORs for overall logistic regression models of prior participation in research

	Any Research *N* = 792		Behavioral Research *N* = 792		Biological Research *N* = 792	
	OR	(95% CI)	OR	(95% CI)	OR	(95% CI)
Race/ethnicity						
African American	2.54*	(1.63–3.94)	2.12*	(1.33–3.36)	2.98*	(1.83–4.84)
Asian American	0.82	(0.46–1.46)	0.75	(0.39–1.43)	0.90	(0.46–1.75)
Latino	1.14	(0.65–1.98)	1.12	(0.61–2.03)	1.07	(0.55–2.08)
White (ref)	–	–	–	–	–	–
Age						
< 65 years (ref)	–	–	–	–	–	–
≥ 65 years	0.65*	(0.47–0.91)	0.81	(0.56–1.17)	0.74	(0.49–1.10)
Marital status						
Single/divorced/widowed (ref)	–	–	–	–	–	–
Married/living with partner	0.67*	(0.45–0.98)	0.55*	(0.36–0.84)	0.63*	(0.41–0.99)
Education						
College graduate or beyond (ref)	–	–	–	–	–	–
Some college	0.79	(0.52–1.19)	0.91	(0.59–1.42)	0.62	(0.38–1.01)
High school or less	0.73	(0.45–1.19)	0.73	(0.42–1.27)	0.67	(0.38–1.17)
Region						
Northern California (ref)	–	–	–	–	–	–
Southern California	1.05	(0.75–1.46)	0.90	(0.63–1.30)	1.20	(0.82–1.76)
Language of interview						
English (ref)	–	–	–	–	–	–
Spanish	0.36*	(0.15–0.85)	0.34*	(0.12–0.92)	0.16*	(0.03–0.72)
Cantonese/Mandarin	0.34	(0.08–1.54)	–	–	0.56	(0.12–2.63)
Insurance						
Private (ref)	–	–	–	–	–	–
Public/government/none	1.17	(0.74–1.85)	1.03	(0.59–1.78)	1.23	(0.71–2.14)
Health literacy						
Low (< 3.5)	0.71	(0.41–1.22)	0.77	(0.41–1.44)	0.59	(0.31–1.14)
Medium to high (≥ 3.5) (ref)	–	–	–	–	–	–
Comorbidities						
0 (ref)	–	–	–	–	–	–
1	1.80	(0.97–3.31)	2.24*	(1.12–4.45)	1.26	(0.60–2.64)
2 or more	2.20*	(1.27–3.80)	2.54*	(1.35–4.77)	2.11*	(1.11–4.04)
Health status						
Poor/fair/good	1.25	(0.87–1.79)	1.46	(0.98–2.17)	1.11	(0.73–1.68)
Very good/excellent (ref)	1.00	–	1.00	–	1.00	–
Gleason score						
1–6	0.83	(0.59–1.18)	0.93	(0.64–1.36)	0.91	(0.61–1.37)
7–10 (ref)	1.00	–	1.00	–	1.00	–
Treatment with hormone therapy or chemotherapy						
Yes	1.17	(0.73–1.89)	0.76	(0.44–1.30)	0.90	(0.51–1.58)
No (ref)	1.00	–	1.00	–	1.00	–

*Note* The total N is the number of participants who had non-missing responses on every covariate included in the model and therefore were included in the analysis

*p < 0.05

With respect to behavioral research, African American men (OR = 2.12, 95% CI 1.33–3.36), those with one comorbidity (OR = 2.24, 95% CI 1.12–4.45), or two or more comorbidities (OR = 2.54, 95% CI 1.35–4.77) were more likely to report participation. Men who were married or living with a partner (OR = 0.55, 95% CI 0.36–0.84) and those who completed the interview in Spanish (OR = 0.34, 95% CI 0.12–0.92) were less likely to report participation in behavioral research.

Similarly, African American men (OR = 2.98, 95% CI 1.83–4.84) and those with two or more comorbidities (OR = 2.11, 95% CI 1.11–4.04) were more likely to report participation in biological/clinical research. Men who were married or living with a partner (OR = 0.63, 95% CI 0.41–0.99) or completed the interview in Spanish (OR = 0.16, 95% CI 0.03–0.72) were less likely to report participation in biological/clinical research.

### Race/ethnicity stratified multivariable analyses

#### Participation in any research

Among African American men, those 65 years and older were less likely to report participation in any research (OR = 0.31, 95% CI 0.14–0.70); and those from the Southern California region were significantly more likely to report participation (OR = 2.43, 95% CI 1.16–5.08; Table 3). Among Asian American men, those who completed the interview in Mandarin or Cantonese were less likely to report participation in any research (OR = 0.21, 95% CI 0.05–0.89). Among Latino men, those who completed the interview in Spanish (OR = 0.27, 95% CI 0.11–0.68) were less likely to report participation in any research. However, those who had public, government, or no insurance (OR = 3.61, 95% CI 1.20–10.89) were more likely to report participation. Among White men, those who were from the Southern California region (OR = 0.50, 95% CI 0.29–0.86) were less likely to report participation in any research. White men reporting excellent or very good health status (OR = 2.03, 95% CI 1.13–3.63) or having two or more comorbidities (OR = 2.66, 95% CI 1.16–6.09) were more likely to report participation.

**Table 3:** Multivariable logistic regression models estimating the effects of patient factors on patient-reported responses to participation in any research, stratified by race/ethnicity

	African American *N* = 148		Asian American *N* = 114		Latino *N* = 187		White *N* = 343	
	OR	95% CI	OR	95% CI	OR	95% CI	OR	95% CI
Age (*ref:* < *65 yrs*)								
≥ 65 years	0.31*	(0.14–0.70)	0.67	(0.21–2.12)	0.56	(0.23–1.40)	1.01	(0.61–1.68)
Marital status (*ref: single/divorced/widowed*)								
Married/living with partner	0.46	(0.20–1.06)	0.62	(0.16–2.36)	1.56	(0.54–4.53)	0.54	(0.29–1.00)
Education (*ref: college graduate or beyond*)								
Some college	0.89	(0.39–2.03)	–	–	0.46	(0.16–1.36)	1.27	(0.66–2.42)
High school or less	0.60	(0.22–1.67)	0.26	(0.03–2.64)	0.34	(0.12–1.00)	1.16	(0.53–2.57)
Language of interview (*ref: English*)								
Spanish			–	–	0.27*	(0.11–0.68)		
Cantonese/Mandarin			0.21*	(0.05–0.89)	–	–		
Region (*ref: Northern CA*)								
Southern California	2.43*	(1.16–5.08)	0.87	(0.28–2.70)	2.02	(0.88–4.66)	0.50*	(0.29–0.86)
Insurance (*ref: private*)								
Public/government/none	1.09	(0.43–2.74)	1.58	(0.46–5.46)	3.61*	(1.20–10.86)	0.49	(0.20–1.17)
Health literacy (*ref: medium to high*)								
Low	0.81	(0.27–2.42)	1.49	(0.36–6.15)	0.69	(0.24–1.94)	0.51	(0.16–1.62)
Health status (*ref: Good/fair/poor)*								
Excellent/very good	0.60	(0.27–1.34)	0.92	(0.29–2.97)	1.51	(0.59–3.91)	2.03*	(1.13–3.63)
Comorbidities (*ref: 0*)								
1	1.48	(0.39–5.59)	1.73	(0.31–9.60)	1.51	(0.43–5.37)	1.84	(0.74–4.59)
2 or more	2.43	(0.74–7.99)	1.19	(0.29–4.89)	1.29	(0.39–4.27)	2.66*	(1.16–6.09)
Gleason score (ref: 1–6)								
7–10	1.07	(0.50–2.28)	0.61	(0.21–1.81)	1.05	(0.42–2.63)	0.63	(0.37–1.05)
Treatment with chemotherapy/hormone therapy (ref: no)								
Yes	0.80	(0.35–1.86)	1.36	(0.16–11.18)	1.88	(0.62–5.73)	1.19	(0.54–2.59)

All models adjusted for all covariates included in the table (except White and African American stratified models were not adjusted for language of interview)

*p < 0.05

#### Participation in behavioral research

Among African American men, participants having a high school education or less (OR = 0.31, 95% CI 0.11–0.90; Table 4) were less likely to report participation in behavioral research. No factors were associated with participation in behavioral research among Asian American men. Among Latino men, those who completed the survey in Spanish were less likely to report participation in behavioral research (OR = 0.26, 95% CI 0.10–0.70). However, Latino men who reported having public, government, or no insurance (OR = 4.17, 95% CI 1.14–15.21) or reported one comorbidity (OR = 8.12, 95% CI 1.04–63.49) were more likely to report participating in behavioral research. And among White men, those who were married or living together (OR = 0.37, 95% CI 0.19–0.70) or were from the Southern California region (OR = 0.51, 95% CI 0.28–0.94) were less likely to report participation in behavioral research. Alternatively, White men who reported excellent or very good health status (OR = 2.36, 95% CI 1.25–4.45) or had two or more comorbidities (OR = 3.28, 95% CI 1.29–8.35) were significantly more likely to report participation in behavioral research.

**Table 4:** Multivariable logistic regression models estimating the effects of patient factors on patient-reported responses to participation in behavioral research, stratified by race/ethnicity

	African American *N* = 148		Asian American *N* = 114		Latino *N* = 187		White *N* = 343	
	OR	95% CI	OR	95% CI	OR	95% CI	OR	95% CI
Age (*ref:* < *65 yrs*)								
≥ 65 years	0.47	(0.21–1.10)	1.62	(0.37–7.14)	0.75	(0.27–2.10)	1.15	(0.66–2.01)
Marital status (*ref: single/divorced/widow*)								
Married/living together	0.43	(0.19–1.00)	0.55	(0.09–3.34)	1.64	(0.49–5.49)	0.37*	(0.19–0.70)
Education (*ref: college graduate or beyond*)								
Some college	0.95	(0.41–2.19)	–	–	0.54	(0.17–1.72)	1.31	(0.66–2.60)
High school or less	0.31*	(0.11–0.90)	0.25	(0.02–3.71)	0.32	(0.10–1.04)	1.65	(0.71–3.82)
Language of interview (*ref: English*)								
Spanish	–	–	–	–	0.26*	(0.10–0.70)	–	–
Cantonese/Mandarin	–	–	–	–	–	–	–	–
Region (*ref: Northern CA*)								
Southern California	2.01	(0.95–4.26)	0.41	(0.08–2.14)	1.49	(0.58–3.82)	0.51*	(0.28–0.94)
Insurance (*ref: private*)								
Public/government/none	1.35	(0.51–3.56)	1.81	(0.44–7.47)	4.17*	(1.14–15.21)	0.40	(0.15–1.06)
Health literacy (*ref: medium to high*)								
Low	0.81	(0.24–2.74)	0.44	(0.05–3.64)	0.92	(0.29–2.93)	0.67	(0.21–2.15)
Health status (*ref: Good/fair/poor)*								
Excellent/very good	0.70	(0.30–1.64)	0.53	(0.13–2.24)	2.81	(0.94–8.41)	2.36*	(1.25–4.45)
Comorbidities (*ref: 0*)								
1	1.01	(0.25–4.19)	1.18	(0.14–9.72)	8.12*	(1.04–63.49)	2.14	(0.77–5.94)
2 or more	2.01	(0.56–7.20)	0.52	(0.11–2.49)	4.93	(0.68–35.81)	3.28*	(1.29–8.35)
Gleason score (ref: 1–6)								
7–10	1.51	(0.68–3.32)	0.55	(0.15–2.09)	1.39	(0.50–3.90)	0.64	(0.37–1.13)
Treatment with chemotherapy/hormone therapy (ref: no)								
Yes	0.66	(0.26–1.66)	0.61	(0.04–8.56)	1.10	(0.31–3.88)	0.60	(0.23–1.57)

All models adjusted for all covariates included in the table (except White and African American stratified models were not adjusted for language of interview)

*p < 0.05

#### Participation in biological/clinical research

Few variables were associated with participation in biological research for all racial/ethnic groups (Table 5). Among African American men, older participants (OR = 0.38, 95% CI 0.17–0.84) were less likely to participate in biological research; and those from Southern California region (OR = 2.21, 95% CI 1.06–4.59) were more likely to participate. No factors were associated with biological research participation among Asian American men. Among Latino men, those with some college education (OR = 0.20, 95% CI 0.04–0.94) or those who completed the interview in Spanish (OR = 0.07, 95% CI 0.01–0.64) were significantly less likely to report participation in biological research. Latino men from Southern California (OR = 4.34, 95% CI 1.15–16.34) were more likely to report participation in biological research compared to Northern California participants. In contrast, White men from Southern California were significantly less likely to report participation in biological research (OR = 0.51, 95% CI 0.27–0.98).

**Table 5:** Multivariable logistic regression models estimating the effects of patient factors on patient-reported responses to participation in biological research, stratified by race/ethnicity

	African American *N* = 148		Asian American *N* = 114		Latino *N* = 187		White *N* = 343	
	OR	95% CI	OR	95% CI	OR	95% CI	OR	95% CI
Age (*ref:* < *65 yrs*)								
≥ 65 years	0.38*	(0.17–0.84)	0.95	(0.28–3.24)	0.51	(0.13–2.04)	1.14	(0.62–2.11)
Marital status (*ref: single/divorced/widow*)								
Married/living together	0.49	(0.22–1.09)	0.26	(0.06–1.09)	3.57	(0.56–22.80)	0.54	(0.27–1.09)
Education (*ref: college graduate or beyond*)								
Some college	0.85	(0.36–2.03)	–	–	0.20*	(0.04–0.94)	0.88	(0.42–1.87)
High school or less	0.99	(0.35–2.81)	0.43	(0.04–5.02)	0.44	(0.09–2.11)	0.47	(0.16–1.37)
Language of interview (*ref: English*)								
Spanish	–	–	–	–	0.07*	(0.01–0.64)	–	–
Cantonese/Mandarin	–	–	0.22	(0.04–1.16)	–	–	–	–
Region (*ref: Northern CA*)								
Southern California	2.21*	(1.06–4.59)	1.51	(0.42–5.40)	4.34	(1.15–16.34)	0.51*	(0.27–0.98)
Insurance (*ref: private*)								
Public/government/none	1.11	(0.42–2.96)	1.73	(0.43–7.00)	4.34	(0.51–37.20)	0.66	(0.24–1.81)
Health literacy (*ref: medium to high*)								
Low	0.65	(0.21–2.07)	2.37	(0.56–10.04)	0.19	(0.02–1.49)	0.42	(0.10–1.78)
Health status (*ref: Good/fair/poor)*								
Excellent/very good	0.82	(0.38–1.80)	0.84	(0.23–3.08)	1.07	(0.30–3.75)	1.44	(0.73–2.83)
Comorbidities (*ref: 0*)								
1	0.82	(0.20–3.29)	1.45	(0.19–11.04)	0.79	(0.13–4.68)	1.67	(0.51–5.43)
2 or more	1.54	(0.48–4.99)	1.89	(0.34–10.50)	1.59	(0.29–8.63)	2.48	(0.83–7.38)
Gleason score (ref: 1–6)								
7–10	0.87	(0.39–1.90)	0.64	(0.18–2.30)	1.09	(0.34–3.54)	0.89	(0.49–1.64)
Treatment with chemotherapy/hormone therapy (ref: no)								
Yes	0.54	(0.22–1.33)	1.72	(0.18–16.80)	1.69	(0.41–6.95)	0.79	(0.31–1.97)

All models adjusted for all covariates included in the table (except White and African American stratified models were not adjusted for language of interview)

*p < 0.05

## Conclusion

Using a population-based survey of a multi-ethnic cohort of men with a history of PCa, this study aimed to assess racial/ethnic disparities in self-reported research participation. Attention to research participation is warranted due to the paucity of research among racial/ethnic minority groups and the limited investigation of participation in different types of research. Our findings indicate that African American men were more likely to report prior research participation (both behavioral and biological) than White men; and Asian American and Latino men did not significantly differ from White men. However, enhanced recruitment efforts, such as tailoring materials, methods and messages [18], may be needed to address identified barriers (e.g., language, lower education, and older age), particularly among men with PCa who have never participated in research. Providing linguistically appropriate materials, and language and culturally concordant interviewers or research coordinators can help increase minority research participation [19, 20].

Factors associated with increased research participation included two or more comorbidities, excellent or very good health status, public/government/no insurance, and living in the Southern California region. While one of the most common reasons patients are excluded from clinical trials is multiple comorbid conditions [21–23], our study found that men with one or more comorbidities were more likely to report research participation, particularly among Latino and White men. This may be due in part to our inclusion of behavioral research, as comorbidities are often not of concern for exclusion. Additionally, patients with comorbidities may have more opportunities to participate in research, as studies are typically geared towards disease and these patients likely engage more frequently with health care systems conducting research. Patients with comorbidities may also be more inclined to participate to improve their health, as previous studies have noted reasons for research participation include the belief that patients in clinical trials receive better health care [15], along with the possibility of benefiting personally and the potential to help others [24, 25]. We also found participants with excellent and very good health status reported greater research participation; however, this was only found among White men reporting participation in any and behavioral research. Although insurance coverage has been reported as a barrier to clinical trial participation in cancer [26], Latino men in our study with public/government/no insurance were more likely to report participation in any and behavioral research. A research study that oversamples Latino men, based in California and linked to a state-funded treatment program for uninsured men with PCa since 2006 [27, 28], may have contributed to our finding. Additionally, living in the Southern California region was associated with greater participation in biological research among Latino men, and biological and behavioral research among African American men. This finding may be due to a larger population of African American and Latino men in this region, compared to Northern California.

Factors associated with lower research participation among individual racial/ethnic groups included older age, being married or living together, having less than a college education, Spanish or Chines language, and living in the Southern California region (for White men). Older age (65 years or older) was associated with less research participation, which is consistent with previous research, despite a persistent awareness of this disparity [29, 30]. Not only is older age the greatest risk factor for cancer, but individuals 65 years and older are a rapidly increasing age group. This lack of participation may be due to upper age cut-offs in research, the likelihood of increasing comorbidities and frailty with age that might hinder participation, and a lack of intentional recruitment efforts [31].

Being married or living with a partner was significantly associated with lower reported participation in all types of research (any, behavioral, and biological) in the overall model. In race-stratified analyses, being married or living together was only significant among White participants for behavioral research. This is an unusual finding as previous studies have noted family members can influence decisions to participate in research and marital status has been a significant predictor of general willingness to participate for male respondents [32, 33]. However, previous studies have reported inconsistent findings [34]. Stryker and colleagues did not find significant associations between research participation and marital status, although their sample was only 24% male [35]. A study in Brazil found 61% of female respondents were significantly influenced by a spouse in the decision to participate in a trial, while 73% of male respondents were not influenced by a spouse [36]. Similarly, a survey in Taiwan found respondents who were married or lived with a significant other were less likely to participate in medical research [37]. These discrepant findings could be due to population and gender differences; however, more research is needed to further clarify this finding.

Lower education (high school or less) was associated with African American participants being less likely to participate in behavioral research, which is similar to previous studies, although results have been mixed [38, 39]. And some college education was associated with Latino participants being less likely to participate in biological/clinical research. This could be due to a lack of knowledge of research (e.g., clinical trials) and lack of explanation of procedures involved in participation. Studies have noted increasing patients’ understanding about research procedures, including clinical trials, and risks and benefits may enhance research participation and improve participants’ informed decisions [39, 40].

Similar to other studies [38, 41], participants who preferred to complete the interview in Spanish or Chinese were less likely to report participation in research. Studies have noted language and cultural differences can be a barrier to research participation [42, 43]. As a result, researchers should consider minimizing language barriers using bilingual materials (including audio-aided consenting information in preferred language), hiring bilingual and culturally concordant research staff, and utilizing translation services, which helps to establish trust and overcome language or dialect barriers [44, 45].

Additionally, the Southern California region was associated with less reported participation in any and all types of research among White participants. This may be due to National Institutes of Health mandates for recruitment efforts among historically under-represented minority populations [46], and that the Southern California region has a larger minority population than Northern California.

Several limitations to our study are worth noting. First, we relied on self-reports of prior research participation which are subject to recall bias and do not capture recruitment efforts. Second, we did not assess preferred language for healthcare interactions specifically, and we did not evaluate limited English proficiency, or use of interpreters during clinical visits; instead we used language preferred for interview as a proxy. Third, we were unable to disaggregate data on either Asian American or Latino participants due to small subgroup sample sizes. This prevented more nuanced analyses that might capture the diversity within racial/ethnic subgroups (e.g., geographic, cultural, and linguistic backgrounds). As a result, we could not determine whether the disparities we found were due to cultural differences in how patients consider research participation. Additionally, while our response rate of 45% is reasonable and fairly similar to other telephone-based surveys [47], it is a bit lower compared to some studies using state cancer registries [48, 49]. This may also reflect lower response rates among men versus women as noted in previous studies [50, 51]. Lastly, our study recruited men from the California Cancer Registry in 2008 with stage I or II PCa (e.g., localized disease), and by the time we completed interviews in 2012, 15% no longer had localized disease, which may not be representative of PCa patients across the United States. Limitations notwithstanding, this study significantly adds to the literature by examining racial/ethnic disparities in self-reported research participation among a multiethnic and multilingual, understudied population. Building upon previous research, this study delves deeper into factors associated with research participation within each racial/ethnic group.

In conclusion, some minority men with PCa report research participation. However, future studies should address factors contributing to lower reports of research participation among minorities—specifically language barriers—and developing targeted efforts to address older minority patients and those with less education.

## Data Availability

Data will be made available on reasonable request.

## References

[1] Siegel RL, Miller KD, Jemal A (2020). Cancer statistics, 2020. CA Cancer J Clin.

[2] Fitzmaurice C, Akinyemiju TF, Al Lami FH (2018). Global, regional, and national cancer incidence, mortality, years of life lost, years lived with disability, and disability-adjusted life-years for 29 cancer groups, 1990 to 2016: a systematic analysis for the global burden of disease study. JAMA Oncol.

[3] DeSantis CE, Siegel RL, Sauer AG (2016). Cancer statistics for African Americans, 2016: progress and opportunities in reducing racial disparities: cancer statistics for African Americans, 2016. CA Cancer J Clin.

[4] Sedarsky J, Degon M, Srivastava S (2018). Ethnicity and ERG frequency in prostate cancer. Nat Rev Urol.

[5] Skolarus TA, Chan S, Shelton JB (2013). Quality of prostate cancer care among rural men in the Veterans Health Administration: quality of rural prostate cancer care. Cancer.

[6] López-Pérez B, Barnes A, Frosch DL (2017). Predicting prostate cancer treatment choices: the role of numeracy, time discounting, and risk attitudes. J Health Psychol.

[7] Spratt DE, Chan T, Waldron L (2016). Racial/ethnic disparities in genomic sequencing. JAMA Oncol.

[8] Spratt DE, Osborne JR (2015). Disparities in castration-resistant prostate cancer trials. J Clin Oncol.

[9] Borno H, Siegel A, Ryan C (2016). The problem of representativeness of clinical trial participants: understanding the role of hidden costs. J Health Serv Res Policy.

[10] Ford ME, Siminoff LA, Pickelsimer E (2013). Unequal burden of disease, unequal participation in clinical trials: solutions from African American and Latino community members. Health Soc Work.

[11] Pariera KL, Murphy ST, Meng J (2017). Exploring willingness to participate in clinical trials by ethnicity. J Racial Ethn Health Disparities.

[12] Sankar PL, Parker LS (2017). The precision medicine initiative’s All of Us research program: an agenda for research on its ethical, legal, and social issues. Genet Med.

[13] Kaplan CP, Nápoles AM, Narine S (2015). Knowledge and attitudes regarding clinical trials and willingness to participate among prostate cancer patients. Contemp Clin Trials.

[14] Palmer NR, Gregorich SE, Livaudais-Toman J (2018). Racial and ethnic differences in prostate cancer survivors’ perceived engagement in treatment decision-making. J Racial Ethn Health Disparities.

[15] Chew LD, Bradley KA, Boyko EJ (2004). Brief questions to identify patients with inadequate health literacy. Fam Med.

[16] Ware JE Jr, Sherbourne CD (1992) The MOS 36-item short-form health survey (SF-36). I. Conceptual framework and item selection. Med Care 30:473–483 1593914

[17] Katz JN, Chang LC, Sangha O (1996). Can comorbidity be measured by questionnaire rather than medical record review?. Med Care.

[18] McDougall GJ, Simpson G, Friend ML (2015). Strategies for research recruitment and retention of older adults of racial and ethnic minorities. J Gerontol Nurs.

[19] Irvine F, Roberts G, Bradbury-Jones C, Liamputtong P (2008). The researcher as insider versus the researcher as outsider: enhancing rigour through language and cultural sensitivity. Doing cross-cultural research.

[20] Fryer CS, Passmore SR, Maietta RC (2016). The symbolic value and limitations of racial concordance in minority research engagement. Qual Health Res.

[21] Unger JM, Hershman DL, Fleury ME (2019). Association of patient comorbid conditions with cancer clinical trial participation. JAMA Oncol.

[22] Chao HH, Mayer T, Concato J et al (2010) Prostate cancer, comorbidity, and participation in randomized controlled trials of therapy. J Investig Med 58:566–568. 10.231/JIM.0b013e3181cf9002 20072029

[23] Townsley CA, Selby R, Siu LL (2005). Systematic review of barriers to the recruitment of older patients with cancer onto clinical trials. J Clin Oncol.

[24] Wendler D, Krohmal B, Emanuel EJ, Grady C, ESPIRIT Group (2008). Why patients continue to participate in clinical research. Arch Intern Med.

[25] Wright JR, Whelan TJ, Schiff S (2004). Why cancer patients enter randomized clinical trials: exploring the factors that influence their decision. J Clin Oncol.

[26] Frank G (2004) Current challenges in clinical trial patient recruitment and enrollment. SoCRA Source 2:30–38. https://clinicaltrials.llu.edu/sites/clinicaltrials.llu.edu/files/docs/current-challenges-in-clinical-trial-patient-recruitment-and-enrollment.pdf

[27] California IMPACT Program. Improving access counseling and treatment for californians with prostate cancer. https://www.california-impact.org. Accessed 10 Apr 2020

[28] Miller DC, Gelberg L, Kwan L (2008). Racial disparities in access to care for men in a public assistance program for prostate cancer. J Community Health.

[29] Murthy VH, Krumholz HM, Gross CP (2004). Participation in cancer clinical trials: race-, sex-, and age-based disparities. JAMA.

[30] Thake M, Lowry A (2017). A systematic review of trends in the selective exclusion of older participant from randomised clinical trials. Arch Gerontol Geriatr.

[31] Lacey RJ, Wilkie R, Wynne-Jones G (2017). Evidence for strategies that improve recruitment and retention of adults aged 65 years and over in randomised trials and observational studies: a systematic review. Age Ageing.

[32] Nelson RM, Merz JF (2002). Voluntariness of consent for research: An empirical and conceptual review. Med Care.

[33] Lee MM, Chamberlain RM, Catchatourian R (1999). Social factors affecting interest in participating in a prostate cancer chemoprevention trial. J Cancer Educ.

[34] Jirka GW, Bisselow KSM, Smith LM, Shonka N (2019). Evaluating the decisions of glioma patients regarding clinical trial participation: a retrospective single provider review. Med Oncol.

[35] Stryker JE, Wray RJ, Emmons KM, Winer E, Demetri G (2006). Understanding the decisions of cancer clinical trial participants to enter research studies: factors associated with informed consent, patient satisfaction, and decisional regret. Patient Educ Couns.

[36] Lobato L, Bethony JM, Pereira FB (2014). Impact of gender on the decision to participate in a clinical trial: a cross-sectional study. BMC Public Health.

[37] Liu H, Li M (2018). Factors influencing the willingness to participate in medical research: a nationwide survey in Taiwan. PeerJ.

[38] Byrne MM, Tannenbaum SL, Glück S (2014). Participation in cancer clinical trials: why are patients not participating?. Med Decis Making.

[39] Kurt A, Kincaid H, Semler L (2018). Impact of race versus education and race versus income on patients’ motivation to participate in clinical trials. J Racial Ethn Health Disparities.

[40] Burns KEA, Magyarody N, Jiang D (2013). Attitudes and views of the general public towards research participation: research participation: public attitudes. Intern Med J.

[41] Huang H-Y, Ezenwa MO, Wilkie DJ (2013). ResearchTracking: monitoring gender and ethnic minority recruitment and retention in cancer symptom studies. Cancer Nurs.

[42] Aristizabal P, Singer J, Cooper R (2015). Participation in pediatric oncology research protocols: racial/ethnic, language and age-based disparities: Disparities in pediatric oncology research. Pediatr Blood Cancer.

[43] Smith A, Agar M, Delaney G (2018). Lower trial participation by culturally and linguistically diverse (CALD) cancer patients is largely due to language barriers. Asia Pac J Clin Oncol.

[44] Aguirre TM, Koehler AE, Joshi A (2018). Recruitment and retention challenges and successes. Ethn Health.

[45] Kue J, Thorburn S, Keon KL (2015). Research challenges and lessons learned from conducting community-based research with the Hmong community. Health Promot Pract.

[46] Freedman LS, Simon R, Foulkes MA (1995). Inclusion of women and minorities in clinical trials and the NIH Revitalization Act of 1993—the perspective of NIH clinical trialists. Control Clin Trials.

[47] Iredell H, Shaw T, Howat P (2004). Introductory postcards: do they increase response rate in a telephone survey of older persons?. Health Educ Res.

[48] Nagler RH, Gray SW, Romantan A (2010). Differences in information seeking among breast, prostate, and colorectal cancer patients: results from a population-based survey. Patient Educ Couns Suppl.

[49] Smith-Gagen J, Cress RD, Drake CM (2010). Quality-of-life an surgical treatments for rectal cancer—a longitudinal analysis using the California Cancer Registry. Psychooncology.

[50] Kaplan CP, Napoles AM, Hwang ES (2011). Selection of treatment among Latina and non-Latina white women with ductal carcinoma in situ. J Womens Health.

[51] Glass DC, Kelsall HL, Slegers C (2015). A telephone survey of factors affecting willingness to participate in health research surveys. BMC Public Health.

